# Necrotising fasciitis as atypical presentation of infection with emerging *Neisseria meningitidis* serogroup W (MenW) clonal complex 11, the Netherlands, March 2017

**DOI:** 10.2807/1560-7917.ES.2017.22.23.30549

**Published:** 2017-06-08

**Authors:** Anne Russcher, Ewout Fanoy, Ger D J van Olden, Antonie D Graafland, Arie van der Ende, Mirjam J Knol

**Affiliations:** 1 Department of Medical Microbiology, Meander Medical Centre, Amersfoort, the Netherlands; 2 Public Health Service Region Utrecht, Zeist, the Netherlands; 3 Centre for Infectious Diseases Control, National Institute for Public Health and the Environment, Bilthoven, the Netherlands; 4 Department of Surgery, Meander Medical Centre, Amersfoort, the Netherlands; 5 Department of Intensive Care, Meander Medical Centre, Amersfoort, the Netherlands; 6 Department of Medical Microbiology and the Netherlands Reference Laboratory for Bacterial Meningitis, Academic Medical Center, Amsterdam, the Netherlands

**Keywords:** meningococcal disease, Emerging or re-emerging diseases, *Neisseria meningitidis*, necrotising fasciitis

## Abstract

In March 2017, a patient with necrotising fasciitis caused by *Neisseria meningitidis* serogroup W (MenW) clonal complex 11 was diagnosed in the Netherlands. Unusual and severe presentations of MenW infections are common in the current European epidemic. In the Netherlands, the incidence of MenW infections increased 10-fold, from an average of 0.03 per 100,000 population in 2002–2014 to 0.29 in 2016. Awareness of atypical presentations enables timely adequate treatment and public health action.

## Case description and microbiological findings

In March 2017 (day 0), a man in his early 60ies consulted his general practitioner (GP) because of a painful, red and swollen ankle since 1 day. Five days before his GP visit, he had experienced a fever that lasted 2 days and was accompanied by nausea and vomiting, from which he recovered spontaneously. The GP diagnosed a first episode of gout and prescribed a non-steroidal anti-inflammatory drug (NSAID). One day later (day 1), the patient visited the emergency department of a local hospital as the redness had spread and now covered his left lower leg up to the knee (Figure). Blistering was present on the ankle. Physical examination also revealed a red, painful area on his right elbow that the patient had been unaware of up until that moment. Prior to this illness, the patient had not travelled abroad, had generally been in good health and had an unremarkable medical history.

**Figure:**
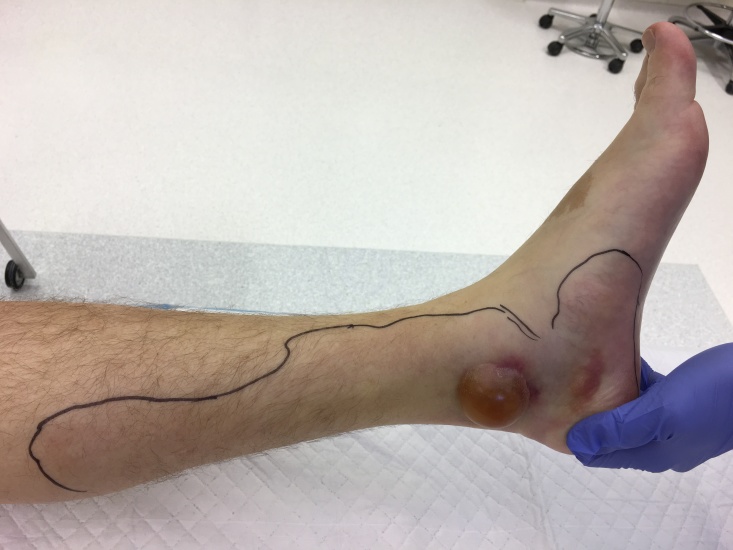
Blister on left ankle as clinical sign in case of necrotising fasciitis due to emerging *Neisseria meningitidis* serogroup W, the Netherlands, March 2017

Emergency surgery was performed immediately because of the clinical suspicion of a necrotising fasciitis. Antibiotic treatment consisting of benzylpenicillin, 20 million units per day (MU/day) intravenously (IV), and clindamycin, 600 mg IV four times daily, was initiated. During surgery, extensive necrosis of subcutis and fascia of the lower leg was present and a fasciectomy of the total lower leg was necessary. Intraoperative Gram-staining of fascia tissue of both the leg and elbow showed the presence of Gram-negative diplococci and ceftriaxone, 2 g IV twice daily, was started.

Post-operatively, the patient was admitted to the intensive care unit with septic shock and received circulatory support with noradrenalin and mechanical ventilation for 48 hours. A second-look operation within 12 hours after the first operation ensured that the first debridement had been sufficient. The following day, tissue cultures were positive for *Neisseria meningitidis*. Identification was performed by Maldi-ToF mass spectrometry (Bruker, Bremen, Germany). Susceptibility testing by E-test (Biomerieux, Marcy l’Etoile, France) with minimal inhibitory concentrations (MIC) in brackets showed susceptibility to penicillin (0.06 mg/L), ceftriaxone (0.04 mg/L), rifampicin (0.032 mg/L) and ciprofloxacin (0.008 mg/L) [1]. Typing at the Netherlands Reference Laboratory for Bacterial Meningitis revealed a serogroup W subtype P1.5,2:F1–1 belonging to the hypervirulent clonal complex 11.

## Treatment and follow-up measures

When results of susceptibility testing became available, the patient was treated with benzylpenicillin, 20 MU/day, later lowered to 12 MU/day for another 7 days. Split skin grafting was performed on his leg. At the time of writing, the patient was recovering well.

The regional public health service (PHS) was contacted the day after admission (day 2) to evaluate the need for prophylactic antibiotic treatment and MenACWY vaccination of close contacts, but such measures were not needed as the patient had lived alone during the week before the onset of disease and there were no close contacts otherwise. The patient received a MenACWY vaccination to prevent a (re)infection in the following 3–5 years [2,3]. The case was notified by the PHS to the National Institute for Public Health and the Environment (RIVM) by the mandatory surveillance system. The Netherlands Early Warning Committee (NEWC) discussed the remarkable clinical presentation of this MenW case and communicated it to Dutch medical professionals via the weekly NEWC report.

## Epidemiological situation in the Netherlands

Before 2015, the incidence of MenW disease was very low in the Netherlands, with an average annual incidence of 0.03 per 100,000 population from 2002 to 2014 (range: 0.01–0.04 per 100,000 population). The incidence increased to 0.05 per 100,000 population (n = 9 cases) in 2015 and 0.29 per 100,000 population (n = 50 cases) in 2016 [4]. The increase started in October 2015 and up until 1 April 2017, 79 MenW cases were reported. In 74 of 79 cases, the finetype could be determined (1 PCR-positive, 73 cultured isolates). It was P1.2,5:F1–1 in 68 of 74 cases (92%). This finetype is associated with hypervirulent clonal complex 11 [5]. Of the remaining five cases (all PCR-positive, culture-negative), not enough material was available to perform typing. MenW incidence was highest among persons aged 65 years or older (0.65/100,000; n = 30), followed by 15–24-year-olds (0.48/100,000; n = 15). Of 79 MenW cases, nine died (11%): four of them were 15–24 years old, two were 45–64 years old and three were 65 years or older. The clinical manifestation was known for 71 cases: 32 had septicaemia (45%), 14 had meningitis (20%), and seven had both septicaemia and meningitis (10%). The other cases had other clinical manifestations including bacteraemic pneumonia (n = 12; 17%), septic arthritis (n = 4; 6%), pericarditis (n = 1) and necrotising fasciitis (n = 1, the case described above). To our knowledge, three patients with septicaemia, one of whom died, presented predominantly with gastrointestinal symptoms. None of the cases were epidemiologically related and there is no geographical clustering.

## Discussion

Infections due to MenW may present differently from the classical clinical presentation of *N. meningitidis* such as meningitis or septicaemia; for example, gastrointestinal presentations have been reported with MenW [6,7]. Necrotising fasciitis can be caused by various bacterial pathogens, but in monomicrobial infections, haemolytic *Streptococci* group A are most commonly identified as the cause [8]. *N. meningitidis* as the causative agent in necrotising fasciitis is extremely rare [9], although there are some reports on severe cellulitis being caused by *N. meningitidis* [10,11].

In the reported case, the presence of infectious foci on both the leg and arm strongly suggests haematogenous spread, probably from nasopharyngeal carriage as no other apparent focus was identified. Blood cultures remained negative, but these were mistakenly drawn after antimicrobial treatment had already been started. The preceding gastrointestinal symptoms could have been related to the MenW infection as was recently described by others [6,7], but they might be a remarkable coincidence as the necrotising fasciitis symptoms did not develop until 3 days later. Reported cases of MenW with gastrointestinal symptoms quickly progressed into septicaemia [7].

Other European countries have also reported MenW cases due to this hypervirulent strain [5,12].

A similar increase in incidence and a similar age distribution in MenW cases has been noted in the United Kingdom (UK) starting in the epidemiological year 2009/2010. Campbell et al. report a case fatality rate of 12% (21/170) in 2014/15 [12]. A similar case fatality rate of 12% (15/129) was reported for the previous period 2010/11-2012/13 (three epidemiological years) [13]. These findings led to emergency vaccination of 13 to 18-year-olds with a MenACWY vaccine [14]. The first results of this programme seem promising, with an observed 69% reduction of predicted cases in the cohort offered vaccination at a vaccination coverage of only 37% [15]. Although this particular case of necrotising fasciitis developed in a patient in their 60ies, introduction of a vaccination programme against MenW in the Netherlands and possibly other European countries might be justified based on the comparable epidemiological pattern and high case fatality rate.

In conclusion, our case underscores the unusual presentation and severity of MenW infections, mostly caused by the hypervirulent clonal complex 11, in the current epidemic. Physicians should be vigilant for rare presentations of MenW infections considering the necessity of prompt diagnosis for optimal treatment. Based on the current epidemiology, inclusion of vaccination against MenW disease in the Dutch national immunisation programme should be discussed.
